# The *TRHDE* and *TSHR* Genes Regulate Laying Traits in Domesticated Zi Geese

**DOI:** 10.3390/cimb47050331

**Published:** 2025-05-04

**Authors:** Xiuhua Zhao, Shan Yue, Yuanliang Zhang, Jinyan Sun, Fugang Peng, Zhenhua Guo

**Affiliations:** Institute of Animal Husbandry, Heilongjiang Academy of Agricultural Sciences, No. 368 Xuefu Road, Harbin 150086, China

**Keywords:** DNA methylation, egg, goose, RNA-seq

## Abstract

Domestic geese are typically seasonal breeders, and the timing and number of eggs they lay vary depending on the region and breed. Previous studies evaluated the Zi goose, which is currently the domestic goose breed with the highest egg production. This research divided the reproductive cycle into four periods and compared the ovarian RNA-seq and DNA methylation data of Zi geese across these time points to identify the key genes that increase egg production. By integrating differentially expressed genes and differentially methylated genes, we identified 525 candidate genes that presented upregulated expression and hypomethylated regions (the hypo-up group). Ultimately, we found that the thyrotropin-releasing hormone degrading enzyme (*TRHDE*) and thyroid-stimulating hormone receptor (*TSHR*) genes play a crucial role in regulating the reproductive cycle of Zi geese. We also generated a proposed model of the relationship between the *TRHDE* and *TSHR* genes in Zi geese. This study provides theoretical references for the development of egg-laying goose breeds and raises additional scientific questions for further discussion among researchers.

## 1. Introduction

Humans have continuously domesticated wild animals as a food source since the emergence of human society [1]. The consumption of eggs as food is predominantly focused on chickens [2]. Artificial selection has led to egg-laying chicken breeds that can lay 250 eggs per year [3], a remarkable figure compared with their avian ancestors. Currently, the meat products commonly consumed by humans include pork, beef, mutton, seafood, turkey, and chicken. However, the variety of edible poultry eggs available is rather limited. Our team has aimed to develop egg-laying goose breeds to produce more goslings and to obtain more eggs [4]. Domestic geese are typically seasonal breeders, and the timing and number of eggs they lay vary depending on the region and breed [5,6,7,8]. Major supermarkets often need to sign stable, long-term supply contracts with vendors, and the current egg-laying situation of domestic geese clearly cannot provide a stable supply of eggs, which severely restricts the development of domestic geese [9]. The Zi goose, which can lay up to 100 eggs per year per bird, is an excellent breed for developing commercially relevant egg-laying goose varieties [4,6].

Regarding the definition of the egg-laying cycle, it can currently be divided into four periods: early laying (EL) [10], peak laying (PL) [11], later laying (LL) [12], and egg ceased (EC) [13]. In Wanxi white geese, comparisons between the laying and egg ceased periods revealed the relationship between miR-144-y and *DIO3* [14]. In Jilin white geese, analyses across the EL, PL and LL suggested that soluble *N*-ethylmaleimide-sensitive factor attachment protein receptor (*SNARE*) might be involved in regulating the sexual behaviour of male geese [15]. Comparisons between the EL, PL and EC in Wanxi white geese identified four genes that regulate egg production [16]. However, there have been no reports on gene expression comparisons across all four periods in domestic geese.

In the past 3 years, there have been numerous reports on genes that regulate egg production in domestic geese. The genes that promote egg-laying include gonadotropin releasing hormone (*GnRH*), follicle-stimulating hormone (*FSH*), luteinising hormone (*LH*), vasoactive intestinal peptide (*VIP*), prolactin (*PRL*), iodothyronine deiodinase 3 (*DIO3*), phosphodiesterase 6C (*PDE6C*), rhodopsin (*RHO*), membrane frizzled-related protein (*MFRP*), prothrombin (*F2*), apolipoprotein B (*APOB*), interleukin-6 (*IL6*), inhibin subunit beta B (*INHBB*), bone morphogenetic protein 5 (*BMP5*), forkhead box L2 (*FOXL2*), chromogranin A (*CgA*, *CHGA*), secreted phosphoprotein 1 (*SPP1*), complement C6 (*C6*), marginal zone B1 (*MZB1*), glycoprotein Ib platelet subunit alpha (*GP1BA*), and Fc gamma binding protein (*FCGBP*) [4,7,14,16,17,18,19,20,21,22,23,24,25,26]. Genes that inhibit egg-laying include cytochrome P450 family 19 subfamily A member 1 (*CYP19A1*) [23]. We considered these genes to be the primary candidates in our study; we also considered other genes mentioned in the Supplementary Materials of the cited studies.

In the present study, we attempted to identify key genes that regulate laying traits in domestic Zi geese by comparing differentially expressed genes (DEGs) and differentially methylated genes (DMGs) across various reproductive periods using RNA-seq and DNA methylation approaches. Considering that the single-nucleotide polymorphism (SNP) of the thyrotropin-releasing hormone-degrading enzyme (*TRHDE*) gene is closely related to the postpartum anoestrus period in Murrah buffalo [27], we also screened for SNPs in key genes among the experimental geese to ensure the accuracy of the results. The findings of this study can provide theoretical references for the development of egg-laying goose breeds.

## 2. Methods

### 2.1. Ethics Statement

The Zi goose research was approved by the Committee for Animal Welfare to the Institute of Animal Husbandry HAAS (No. NKY-20140506).

### 2.2. Animals

**Figure 1:**
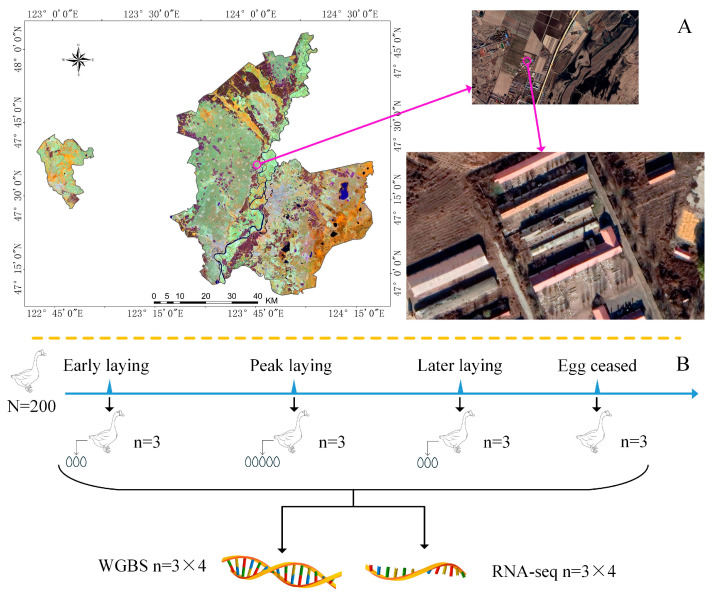
Geographic location and experimental design. (**A**) The precise location of the farm is at 47.27°N, 123.69°E. White Zi geese can be seen in the exercise yard. This study used the third building. (**B**) Four time points were selected during the reproductive cycle of Zi geese to collect experimental samples, which were used for haematoxylin–eosin staining, DNA methylation detection, and RNA-seq analysis.

The experimental Zi geese were farmed on the Heilongjiang Academy of Agricultural Sciences experimental farm (Fulaerji District, Qiqihar City, 47.27N, 123.69E; Figure 1A). Geese from different locations have completely different egg-laying results [28,29]. The experimental site was precisely located using ArcGIS Desktop (ArcMap) software version 10.8.2 (ESRI, Inc., Redlands, CA, USA). The average annual temperature ranged from −2 °C to 11 °C, with a daylight duration ranging from 10 to 15 h. A total of 200 Zi geese were used in this study. The goslings were hatched on 10 July 2023, with a flock composed of 160 females and 40 males. The rearing methods followed those described in previous studies [4]. In short, all geese were raised under natural temperature and lighting conditions. During the day, they freely roamed in the exercise yard with access to feed and water, while at night, they were housed in a room without artificial lighting. The feed contained 11.3 MJ/kg of energy, 2.35% calcium, 0.75% lysine, and 15.5% crude protein.

### 2.3. Egg-Laying Measurements and Sample Collection

Based on the egg-laying patterns of Zi geese reported in 2023 [4], the number of eggs laid by the geese was recorded continuously starting on 1 March 2024. The egg-laying data collected every day is the total number of eggs collected at the sports field and egg-laying nest during each daytime. The daily egg-laying rate is the number of eggs laid per day divided by the total number of female geese in the entire group. According to the laying rate of the flock, four key time points were selected during the egg-laying process: EL (16 March 2024), PL (17 May 2024), LL (12 July 2024), and EC (27 August 2024). Figure 1B shows that at each time point, three healthy female geese were selected. After ensuring animal welfare, the selected geese were slaughtered, and their ovary weights and body weights were measured for comparison with one-way analysis of variance (ANOVA). Ovaries with follicles larger than 0.3 cm in diameter were removed and divided into three parts: one for haematoxylin–eosin (HE) staining, one for DNA methylation analysis, and one for RNA-seq analysis. During EL, PL and LL, the female geese were selected based on their egg-laying behaviour. In summary, the ovarian tissues for DNA methylation and RNA-seq analysis were frozen in liquid nitrogen and sent to the testing company. The ovarian tissues for histological sections were cut into small pieces of approximately 3 mm in size, with a tissue block volume to 4% paraformaldehyde ratio of 1:7. After delivery to the laboratory, the tissues were dehydrated, embedded in paraffin wax, sectioned, and stained with HE [30,31].

### 2.4. RNA-Seq Data Analysis

Messenger RNA (mRNA) from ovarian samples was isolated from total RNA using poly-T oligo-attached magnetic beads. Subsequently, sequencing was performed using the HiSeq™ X TEN platform (Illumina, San Diego, CA, USA). Initially, the gene expression levels of the 12 samples were measured using fragments per kilobase of transcript per million fragments mapped (FPKM) values, and Pearson correlation coefficients were calculated to determine the pairwise correlations among all samples. Next, principal component analysis (PCA) was conducted on the 12 samples. The purpose of these two steps was to confirm the correctness of the sample grouping. Finally, DEGs in the four groups (*n* = 12) were identified, with the criteria of log2 (fold change) > 1 and false discovery rate (FDR) < 0.05 used to screen for DEGs.

### 2.5. Gene Ontology (GO) and Kyoto Encyclopedia of Genes and Genomes (KEGG) Pathway Enrichment Analyses

The DEGs between PL and EC were extracted from the RNA-seq data. Data from the 12 samples were subjected to GO and KEGG enrichment analyses. The topGO package (version 2.26.0) in R (version 4.2.3) was used for GO enrichment analysis. KEGG enrichment analysis was conducted using the online analysis tool KOBAS (www.kobas.cbi.pku.edu.cn, accessed on 21 January 2025) [8]. Items with *p* < 0.05 were defined as significantly enriched.

### 2.6. DNA Methylation Library Construction, Sequencing, and Raw Data Processing

DNA isolated from ovarian samples was sequenced with the HiSeq™ X TEN platform (Illumina, San Diego, CA, USA). Twelve samples with a sequencing coverage depth of over 5× were extracted for methylated C sites in different sequence contexts (CG, CHH, and CHG, where H represents A, C, or T). The methylation levels in various gene functional regions were compared among the four periods (early laying, peak laying, later laying, and egg ceased) and plotted for analysis. The average methylation levels of C sites in different genomic functional regions (2 kilobase pairs [kb] upstream, exon, intron, and 2 kb downstream) were analysed. Subsequently, PCA was conducted on the 12 samples to confirm the correctness of the grouping. The criteria for screening DMGs were set as an average methylation level difference between groups >0.2 and *p* < 0.05.

### 2.7. Combination of DEGs and DMGs

Based on the analysis results, the data of DEGs and DMGs were integrated to identify genes potentially involved in reproductive regulation. Data from PL and EC were extracted to construct a Venn diagram [32]. Genes with downregulated expression and hypermethylated regions (hyper-down) were considered to be one dataset, while genes with upregulated expression and hypomethylated regions (hypo-up) were considered another dataset. These datasets were prioritised for in-depth analysis as a pool of candidate genes [33].

### 2.8. Screening Key Genes Regulating Laying Traits in Zi Geese

**Figure 2:**
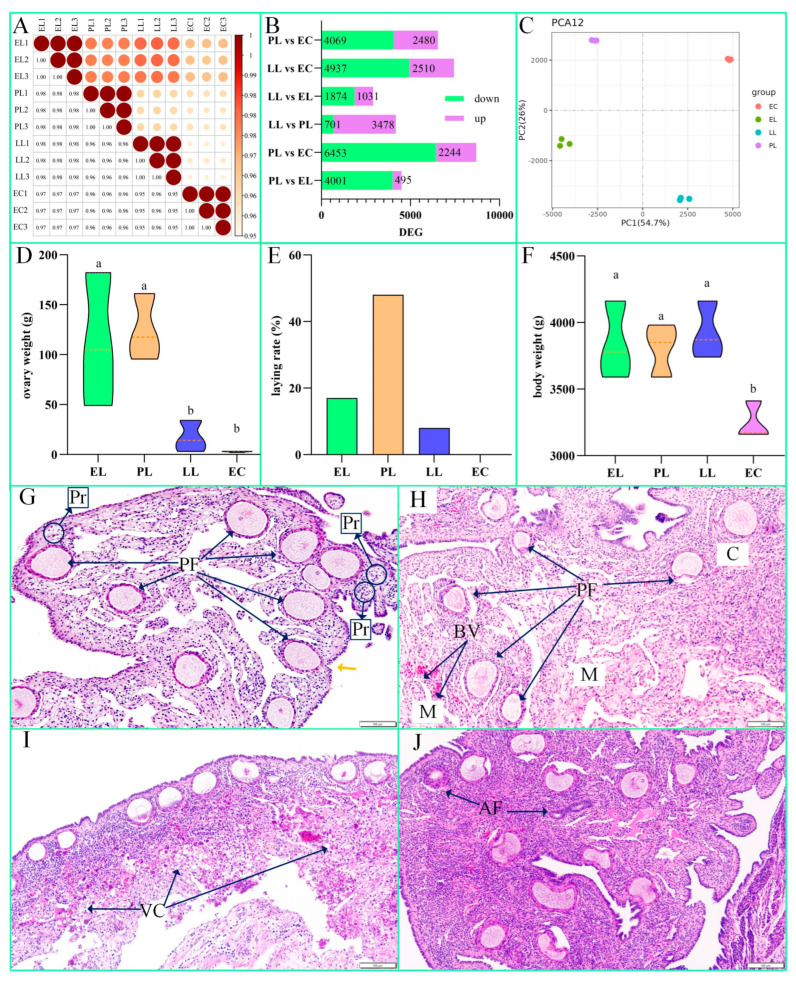
RNA-seq and ovarian changes. (**A**–**C**) The ovarian RNA expression, differentially expressed genes (DEGs), and principal component analysis (PCA) results, respectively, for the 12 samples across four periods: early laying (EL), peak laying (PL), later laying (LL), and egg ceased (EC). (**D**–**F**) The ovarian weight, laying rate, and body weight, respectively, across the four periods. Different letters indicate significant differences (*p* < 0.05). (**G**–**J**) The haematoxylin–eosin (HE) staining results of ovaries during the EL, PL, LL, and EC periods, respectively. The following are marked in the images: cortex (C), medulla (M), blood vessels (BV), primordial follicles (Pr), primary follicles (PF), vacuolated cells (VC), and atretic follicles (AF). The scale bar in the figure is 100 µm.

Based on previous studies, the data from 12 geese were combined to construct a heatmap of DEGs using log10 (FPKM) values, with the parameter scale = row. A total of 25 candidate DEGs were identified, including *PRL*, *VIP*, thyrotropin-releasing hormone (*TRH*), *FSH*, insulin-like growth factors (*IGF1* and *IGF2*), and neuropeptide Y (*NPY*). These genes were differentially expressed in at least one of the comparison groups (a total of six comparison groups, see Figure 2B and Figure S1).

### 2.9. Protein–Protein Interaction (PPI) Network Construction

DEGs related to laying traits, including *PRL*, *VIP*, *TRH*, *FSH*, *IGF1*, *IGF2*, and *NPY*, were extracted from PL and EC. The PPI network for these DEGs was constructed using the STRING database (version 11.5) [34].

### 2.10. Analysis of SNPs and Insertion/Deletions (InDels) in the TRHDE and Thyroid-Stimulating Hormone Receptor (TSHR) Genes

The *TRHDE* and *TSHR* gene expression data from the 12 samples were aligned to the reference genome to obtain SNP and InDel information. Specifically, the mpileup command from samtools [35] was used to generate a.bcf file, and VarScan [36] was employed to identify all possible SNP and InDel sites.

### 2.11. Molecular Docking of the TRHDE Protein with TRH

The amino acid sequence of TRHDE (XP_013053329.1) and the structure of TRH (PubChem CID131841495) were obtained from the National Center for Biotechnology Information (NCBI, www.ncbi.nlm.nih.gov, accessed on 21 January 2025). Docking simulations were performed based on the mutation sites in the exons identified through SNP analysis. The molecular structures of TRHDE were optimised using the SWISS-MODEL tool (https://swissmodel.expasy.org/, accessed on 21 January 2025). The three-dimensional (3D) TRHDE protein structures were processed using the PyMOL (version 2.6) software to remove water molecules. Molecular docking was performed using AutoDock Tools (version 2.6). The results were imported into the PyMOL software for visualisation and analysis [37].

## 3. Results

### 3.1. RNA-Seq Analysis

Figure 2A, 2B and 2C show the correlation of ovarian RNA expression, DEGs, and the PCA results, respectively, for the 12 samples across four periods: early laying, peak laying, later laying, and egg ceased. The differences within each group were small, while the differences between the groups were more pronounced (Figure 2A). Figure 2B shows that the comparison between PL and EC had the most significant gene expression differences, with a total of 6453 downregulated genes and 2244 upregulated genes. As shown in Figure 2C, the 12 samples were divided into three distinct groups. These results indicate that the differences among the four periods are significant, and the reliability of the subsequent analysis is high. Figure S1 shows the DEGs. Of note, Figure S1E indicates that in the comparison between PL and EC, both *TRHDE* and *TSHR* were significantly upregulated. In fact, after completing the entire analysis, we identified *TRHDE* and *TSHR* as key genes regulating reproduction in Zi geese; these genes are marked on the volcano plot.

### 3.2. Analysis of Ovarian Changes During Goose Egg Laying

Figure 2D, 2E and 2F show the ovarian weight, laying rate, and body weight, respectively, across the four periods. During the EC, the ovarian and body weights were significantly lower than those during EL and PL (Figure 2D,F). The laying rate was highest during PL (75 eggs, 157 female geese), and, as expected, egg laying had stopped during EC (0 eggs, 150 female geese) (Figure 2E). Figure 2G, 2H, 2I and 2J present the histological results of ovaries stained with HE during the four periods, respectively. In Figure 2G (EL), primordial follicles and primary follicles can be observed. The orange arrow indicates a special section that will undergo ovulation upon maturation. In Figure 2H (PL), follicles with diameters larger than 3 mm were removed before sectioning. There is an evident reduction in the number of primordial and primary follicles, with a prominent medulla and abundant blood vessels. In Figure 2I (LL), post-ovulatory granulosa and thecal cells regress through orderly apoptosis, and numerous vacuolated cells are present. In Figure 2J (EC), the number of atretic follicles increases. Follicles that develop during this reproductive cycle gradually regress and begin preparations for the next reproductive cycle.

### 3.3. GO and KEGG Pathway Enrichment Analyses

**Figure 3:**
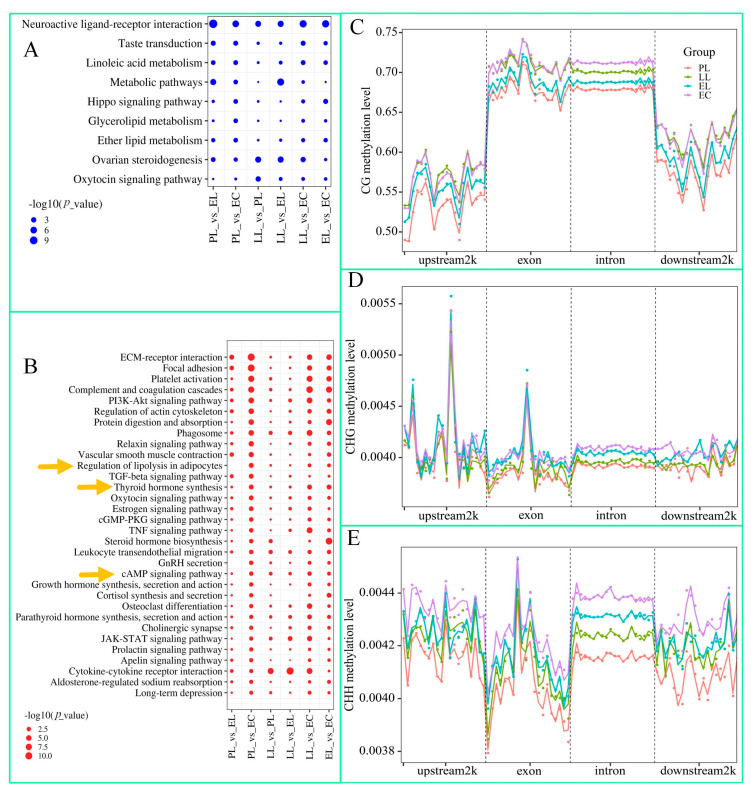
Kyoto Encyclopedia of Genes and Genomes (KEGG) pathway enrichment and methylation level maps of genomic functional elements across the four periods. (**A**,**B**) The KEGG pathway enrichment results between PL and EC for the 12 samples for the downregulated and upregulated genes, respectively. The orange arrows indicate the cAMP signalling pathway, regulation of lipolysis in adipocytes, and thyroid hormone synthesis. (**C**–**E**) The ovarian DNA methylation levels of the 12 samples across the four periods: CG, CHG, and CHH, respectively. Abbreviations: EL, early laying period, PL, peak laying period, LL, later laying period, and EC, egg ceased period.

Figure 3A,B show the KEGG pathway enrichment of RNA-seq data in the 12 samples. The pathways of downregulated genes between PL and EC do not involve the *TRHDE* and *TSHR* genes (Figure 3A). The upregulated genes include *TSHR*, which is enriched in the cAMP signalling pathway, regulation of lipolysis in adipocytes, and thyroid hormone synthesis (Figure 3B). Figure S2 shows the GO enrichment results; *TRHDE* and *TSHR* are not included in either downregulated (Figure S2A) or the upregulated genes (Figure S2B).

### 3.4. DNA Methylation Analysis

Analysis of methylation levels across gene functional regions in the four groups revealed that the methylation level in the gene body (exon and intron) was highest in the CG sequence context, while methylation levels 2 kb upstream and 2 kb downstream of the gene were relatively lower (Figure 3C). However, differences in methylation levels among the groups were not significant in the CHH and CHG sequence contexts (Figure 3D,E). The PCA results of DMGs are shown in Figure S2. Notably, based on the CG sequence context, the data from the 12 geese could be grouped according to the four periods (Figure S2C); this grouping did not occur for the CHH and CHG sequence contexts (Figure S2D,E).

### 3.5. Screening Key Genes That Regulate Egg-Laying Traits in Zi Geese

**Figure 4:**
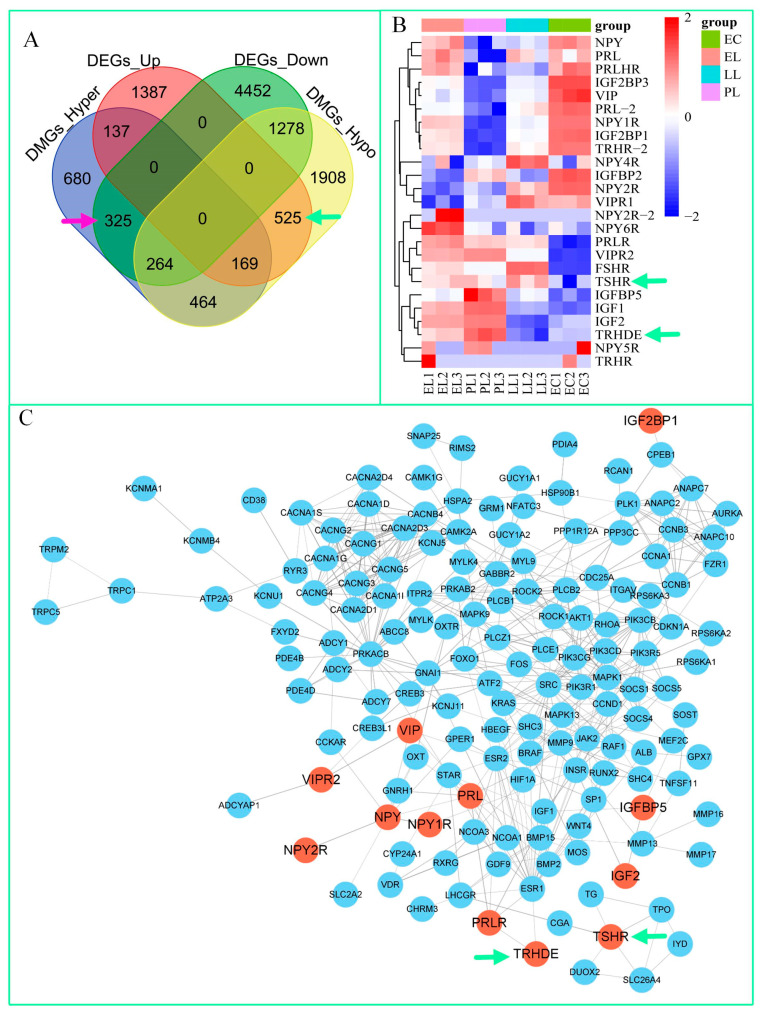
Key genes regulating laying traits in Zi geese. (**A**) Venn diagram of differentially expressed genes (DEGs) and differentially methylated genes (DMGs) between the peak laying (PL) and egg ceased (EC) periods. The green and purple arrows indicate the candidate gene pools screened in this study. (**B**) Heatmap of DEGs for the 12 individual geese across the early laying (EL), PL, later laying (LL), and EC periods. (**C**) Protein–protein interaction network. Red represents the key DEGs for PL_vs_EC, including *PRL*, *VIP*, *TRH*, *FSH*, *IGF1*, *IGF2* and *NPY*, while blue represents other egg production–related DEGs for PL_vs_EC.

Figure 4A shows the Venn diagram of DEGs and DMGs between PL and EC. We identified 525 upregulated genes with hypomethylated regions (hypo-up); they served as the sample pool for screening candidate genes in this study. These 525 genes include *TRHDE* and *TSHR* (indicated by green arrows). In addition, we found 325 downregulated genes with hypermethylated regions (hyper-down), which we used as references (indicated by purple arrows). Figure 4B displays the heatmap of DEGs for the 12 individual geese. It lists 25 candidate genes related to reproductive performance in geese, including *TRHDE* and *TSHR* (indicated by green arrows).

### 3.6. PPI Network Construction

Figure 4C shows the PPI network in which the genes of particular focus in this study are marked in red, and the TRHDE and TSHR proteins are indicated by green arrows. These two proteins have relatively few connections with other proteins. This is similar to the results of GO and KEGG pathway enrichment analyses, where TSHR has more connections with other proteins than TRHDE. However, in the overall PPI network, both proteins are considered to be peripheral.

### 3.7. SNP and InDel Analysis of the TRHDE and TSHR Genes

**Figure 5:**
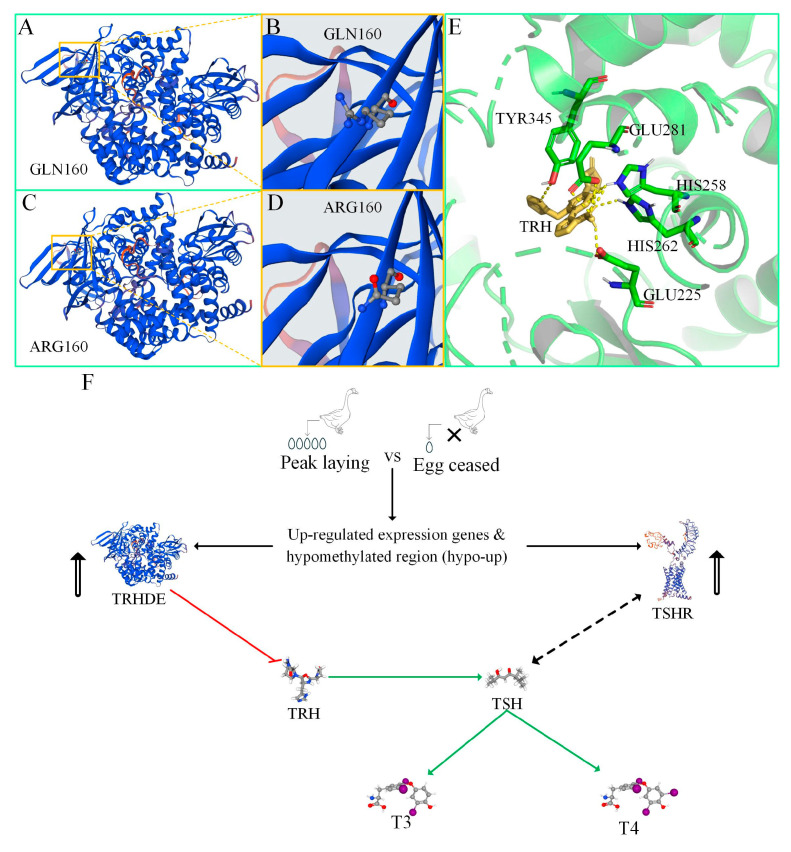
Molecular docking of the TRHDE protein with TRH. (**A**–**D**) The panels present the three-dimensional structure of the wild-type (GLN160) and mutant (ARG160) forms of the TRHDE. (**A**,**B**) A close-up view of GLN160; (**C**,**D**) a close-up view of ARG160. (**E**) The binding pocket of TRHDE with TRH, with yellow dashed lines representing the binding interactions. (**F**) The proposed model of the relationship between *TRHDE* and *TSHR* involving triiodothyronine (T3) and thyroxine (T4).

Table S1 shows the SNPs of the *TRHDE* and *TSHR* genes. There are no SNPs in the exons of the *TSHR* gene among the six geese. The majority of the *TRHDE* SNPs are located in the 3′ untranslated region (UTR), with only one missense SNP (479A>G) in the exon that leads GLN160 change to ARG160 (Figure 5A–D). Additionally, we identified four synonymous variants.

### 3.8. Molecular Docking of TRHDE with TRH

Figure 5A–D compares the 3D structures of wild-type (GLN160) and mutant-type (ARG160) TRHDE. The mutation of amino acid 160 does not alter the overall structure of TRHDE. Additionally, Figure 5E shows the binding pocket of TRHDE with TRH. Five amino acids are involved in the binding: TYR345, GLU281, HIS258, HIS262, and GLU225. Amino acid 160 is not involved in the binding pocket. Therefore, the SNP does not affect the results of this study.

## 4. Discussion

Figure 5F summarises the key genes identified for regulating egg-laying traits in domestic Zi geese and proposes a model of the relationship between *TRHDE* and *TSHR* genes. When comparing the DEGs and DMGs between PL and EC, we found that both *TRHDE* and *TSHR* are upregulated with hypomethylated regions (hypo-up). *TRHDE* inhibits TRH, which in turn promotes thyroid-stimulating hormone (*TSH*), whose receptor is encoded by *TSHR*. *TSH* promotes the secretion of triiodothyronine (T3) and thyroxine (T4). Studies have reported that, through light regulation, the serum T3 and T4 levels in high-yielding groups are higher than those in low-yielding groups [8,24,38]. These findings indirectly support our proposed model of the relationship between *TRHDE* and *TSHR*.

Light exposure has been shown to downregulate the expression of *TSHR* and *TRH* in the pituitary of Magang ganders [39]. By cloning the *TSHR* gene from domestic geese, researchers found that the TSHR protein in geese is closely related to other avian species, especially ducks and chickens [40]. However, based on the available references, there is no direct evidence indicating that *TSHR* is associated with reproduction in domestic geese. Similarly, there have been no reports linking *TRHDE* to reproduction in domestic geese.

In the context of artificial light induction for off-season breeding in Yangzhou geese, high expression of *TSH* can promote egg-laying [38]. In the present study, we observed differences in *TRHDE* and *TSHR* expression across the four periods (Figure S1). Moreover, the four distinct reproductive periods can be clearly distinguished (Figure S1), which lays a solid foundation for subsequent analyses.

Gene regulation of egg production in domestic geese varies across breeds. In Wanxi white geese, the hypothalamus increases egg production through the high expression of *PDE6C*, *RHO*, *MFRP*, *F2*, *APOB*, and *IL6* [7]. However, a comprehensive analysis of the hypothalamic–pituitary–gonadal axis suggests that miR-144-y and *DIO3* subtly regulate egg production in Wanxi white geese [14]. In Zhedong white geese, gene expression analysis of the hypothalamic–pituitary–gonadal axis indicates that *GnRH* is a key gene regulating egg production [24]. In Zhedong white geese regulated by light exposure, the ovaries of the high-yielding group highly express *SPP1*, *C6*, *MZB1* and *GP1BA*, while small white follicles highly express *SPP1*, angiopoietin-like 5 (*ANGPTL5*), and cholinergic receptor nicotinic alpha 4 (*CHRNA4*) [26]. We focused on ovarian gene expression and DNA methylation, with a particular emphasis on the 525 candidate hypo-up genes (Figure 4A). Another important gene that regulates ovarian development is *FSHR*. The development of follicles in goslings is regulated by *FSHR*. A comparative analysis of goslings aged 1 to 6 weeks revealed two peaks of *FSHR* expression at 2 and 4 weeks, with a decrease to the lowest level by the sixth week [41]. *FSHR* inhibits ovulation in Zhedong White geese primarily by suppressing the development of pre-ovulatory follicles [42], which is consistent with our research findings that show the expression level of *FSHR* is at its lowest during PL.

Given the large number of candidate genes we identified, we suggest that egg production in domestic geese is a complex quantitative trait regulated by multiple genes and influenced by environmental factors and epigenetic modifications. Moreover, there are significant differences in gene expression among domestic goose breeds [6,16], which may account for the varying reports of genes that regulate egg production across studies [4,7,14,16,17,18,19,20,21,22,23,24,25,26]. In our previous research, we identified the *NPY* gene as a key regulator of egg production in Sanhua geese under light regulation [8]. In contrast, based on the present study, we propose that *TRHDE* and *TSHR* play a crucial role in regulating the reproductive cycle of Zi geese. *PRL* is a key gene that has been frequently reported to regulate egg laying in geese. A study on the ovarian miRNA-mRNA interactions in Wanxi White geese found that, compared to the pre-laying period, the expression of *PRL* was significantly downregulated during the laying period [16]. This is consistent with the results in Figure 2B. Furthermore, through light regulation to induce off-season breeding in Zhedong White geese, it was found that the expression level of the *PRL* gene in the control group was significantly lower than that in the off-season breeding group [24].

Relatively speaking, the conclusions regarding the impact of serum hormone levels on egg production in domestic geese have been more consistent compared with the impact of gene expression. Studies have shown that in Zhedong white geese, the serum T3 and T4 levels are significantly higher in the off-season breeding group than in the normal group [24,38]. In our previous research on the effect of light on egg production in Sanhua geese, we also detected that during PL, the T3 and T4 levels are significantly higher in the high-yielding group than in the low-yielding group [8].

The impact of SNPs on reproduction is significant, with the classic example being the *GDF9* SNP in sheep. The mutation type G8 FecGH (S395F) significantly increases litter size in heterozygotes; however, homozygotes for this mutation become infertile, making it very difficult to eliminate such breeding sheep using traditional breeding methods [43]. In the Sichuan white goose population, the SNP in exon 9 of the *ATAPA1* gene plays a crucial role in the number of eggs laid. The wild type lays fewer than 48 eggs, while the mutation type exceeds 79 eggs [20]. It is unclear whether the *TRHDE* SNP located in the 3′-UTR affects the promoter of this gene. Additionally, the SNP analysis based on only six geese is not representative. We aimed to exclude the potential impact of SNPs on the experimental results. Previous studies have identified SNPs in the 3′-UTR of the *TRHDE* exon and established their correlation with postpartum anoestrus in Murrah buffaloes. Protein docking models have also suggested that the C allele, which changes glutamine to histidine at amino acid 148 of TRHDE, can enhance the stability of TRHDE [27]. In the comparison between PL and EC, amino acid 160 of the TRHDE protein did not participate in the binding pocket. Therefore, the SNP differences in the *TRHDE* and *TSHR* genes do not affect the results of this study (Figure 5A–E).

## 5. Conclusions

By comparing the ovarian DEGs across four periods—early laying, peak laying, later laying, and egg ceased—and integrating the analysis of genomic functional element methylation, we found that the *TRHDE* and *TSHR* genes play a crucial role in regulating the reproductive cycle of Zi geese. However, our research has led to more questions: how do the SNPs present in the domestic goose population affect egg production? During PL, the expression levels of both *TRHDE* and *TSHR* genes increase simultaneously; how do these two genes interact with each other? These questions will be the focus of our future research. On the one hand, this study provides theoretical references for the development of egg-laying goose breeds. On the other hand, it raises more scientific issues for further discussion among researchers.

## Data Availability

Please contact the author to request the data.

## Supplementary Materials

The following supporting information can be downloaded at: https://www.mdpi.com/article/10.3390/cimb47050331/s1.

## Abbreviations

The following abbreviations are used in this manuscript:

Early laying (EL)
Peak laying (PL)
Later laying (LL)
Early ceased (EC)
Thyrotropin-releasing hormone degrading enzyme (*TRHDE*)
Thyroid-stimulating hormone receptor (*TSHR*)
Gonadotropin releasing hormone (*GNRH*)
Follicle-stimulating hormone (*FSH*)
Luteinising hormone (*LH*)
Vasoactive intestinal peptide (*VIP*)
Prolactin (*PRL*)
Iodothyronine deiodinase 3 (*DIO3*)
Phosphodiesterase 6C (*PDE6C*)
Rhodopsin (*RHO*)
Membrane frizzled-related protein (*MFRP*)
Prothrombin (*F2*)
Apolipoprotein B (*APOB*)
Interleukin-6 (*IL6*)
Inhibin subunit beta B (*INHBB*)
Bone morphogenetic protein 5 (*BMP5*)
Forkhead box L2 (*FOXL2*)
Chromogranin A (*cga*, *CHGA*)
Secreted phosphoprotein 1 (*SPP1*)
Complement C6 (*C6*)
Marginal zone B1 (*MZB1*)
Glycoprotein Ib platelet subunit alpha (*GP1BA*)
Fc gamma binding protein (*FCGBP*)
Cytochrome P450 family 19 subfamily A member 1 (*CYP19A1*)
Soluble *N*-ethylmaleimide-sensitive factor attachment protein receptor (*SNARE*)
Differentially expressed genes (DEGs)
Differentially methylated genes (DMGs)
Single nucleotide polymorphism (SNP)
Analysis of variance (ANOVA)
Haematoxylin–eosin (HE)
Messenger RNA (mRNA)
Fragments per kilobase of transcript per million fragments mapped (FPKM)
Principal component analysis (PCA)
False discovery rate (FDR)
Gene Ontology (GO)
Kyoto Encyclopedia of Genes and Genomes (KEGG)
Thyrotropin-releasing hormone (*TRH*)
Insulin-like growth factors (*IGF1* and *IGF2*)
Neuropeptide Y (*NPY*)
Protein–protein interaction (PPI)
Thyroid-stimulating hormone receptor (*TSHR*)
Three-dimensional (3D)
Thyroid-stimulating hormone (*TSH*)
Triiodothyronine (T3)
Thyroxine (T4)
Angiopoietin-like 5 (*ANGPTL5*)
Cholinergic receptor nicotinic alpha 4 (*CHRNA4*)
