# Supplementary material for: The TRHDE and TSHR Genes Regulate Laying Traits in Domesticated Zi Geese
Source: Curr Issues Mol Biol. 2025 May 4;47(5):331. doi: 10.3390/cimb47050331 (PMC12110115; doi:10.3390/cimb47050331)
Supplement: Supplementary file 1 [file cimb-47-00331-s001.zip › Supplementary table S1.pdf]

Supplementary table S1 Sample characteristics SNP of *TRHDE* and *TSHR* gene.

Gene ID	Cds.mutation	Effect variant	Protein_mutation	PL1	PL2	PL3	EC1	EC2	EC3
TRHDE	7026G>A	3_prime_UTR		0;0	1;1	0;0	0;0	0;0	0;0
TRHDE	6920A>T	3_prime_UTR		0;0	0;1	0;0	0;0	0;0	0;0
TRHDE	6771G>A	3_prime_UTR		0;0	0;1	0;0	0;0	0;0	0;0
TRHDE	6163G>A	3_prime_UTR		0;0	1;1	0;0	0;0	0;0	0;0
TRHDE	3832G>A	3_prime_UTR		0;0	0;1	0;0	0;0	0;0	0;0
TRHDE	2095G>A	3_prime_UTR		1;1	1;1	1;1	0;0	0;0	0;0
TRHDE	1838T>C	3_prime_UTR		1;1	1;1	0;1	0;0	0;0	0;0
TRHDE	1776G>A	3_prime_UTR		0;1	1;1	0;1	0;0	0;0	0;0
TRHDE	1661T>A	3_prime_UTR		0;1	0;1	1;1	0;0	0;0	0;0
TRHDE	1354A>G	3_prime_UTR		1;1	1;1	1;1	0;0	0;0	0;0
TRHDE	947A>G	3_prime_UTR		1;1	1;1	1;1	1;1	0;0	0;1
TRHDE	467C>T	3_prime_UTR		0;1	1;1	0;0	0;0	0;0	0;0
TRHDE	153G>T	3_prime_UTR		0;0	0;0	0;1	0;0	0;0	0;0
TRHDE	118T>C	3_prime_UTR		0;0	1;1	1;1	0;0	0;0	0;0
TRHDE	2367T>A	synonymous	789Thr> Thr	1;1	1;1	0;1	0;0	0;0	0;0
TRHDE	2229T>C	synonymous	743His> His	1;1	1;1	0;1	0;0	0;0	0;0
TRHDE	1917C>A	synonymous	639Ala> Ala	0;0	0;1	0;0	0;0	0;0	0;0
TRHDE	1860A>T	synonymous	620Ser> Ser	0;0	0;1	0;0	0;0	0;0	0;0
TRHDE	12460C>T	intron		0;0	0;0	0;0	0;0	0;0	0;1
TRHDE	12468A>G	intron		0;0	0;0	0;0	0;0	0;0	0;1
TRHDE	12474A>G	intron		0;0	0;0	0;0	1;1	1;1	1;1
TRHDE	479A>G	missense	160 Gln> Arg	1;1	1;1	0;0	0;0	0;0	0;0
TSHR	6312C>A	intron		0;0	0;0	0;0	0;1	0;0	0;0
TSHR	5744G>A	intron		0;0	0;0	0;0	0;0	0;1	0;1
TSHR	2855A>G	intron		0;0	0;0	0;0	0;0	0;1	0;0

peak laying period (PL), egg ceased period (EC). 0; 0 represents no mutation in both positive and negative DNA strands, 0; 1 represents a single stranded mutation, 1; 1 represents a double stranded mutation. N = 6. All geese are female.
