# Supplementary figures and images for: The TRHDE and TSHR Genes Regulate Laying Traits in Domesticated Zi Geese
Source: Curr Issues Mol Biol. 2025 May 4;47(5):331. doi: 10.3390/cimb47050331 (PMC12110115; doi:10.3390/cimb47050331)

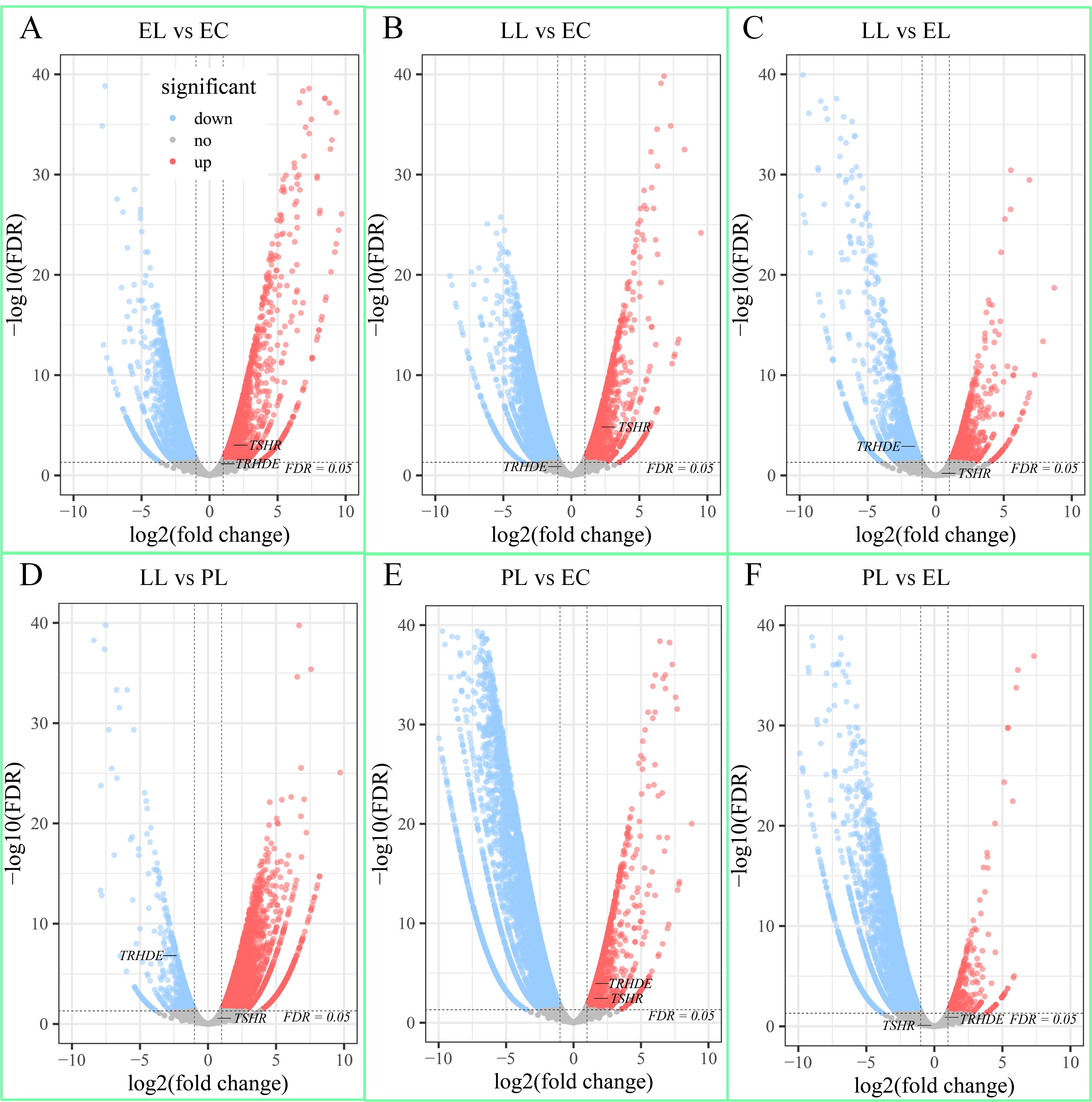

Supplement: Supplementary file 1 [file cimb-47-00331-s001.zip › Supplementary Figure S1.jpg]

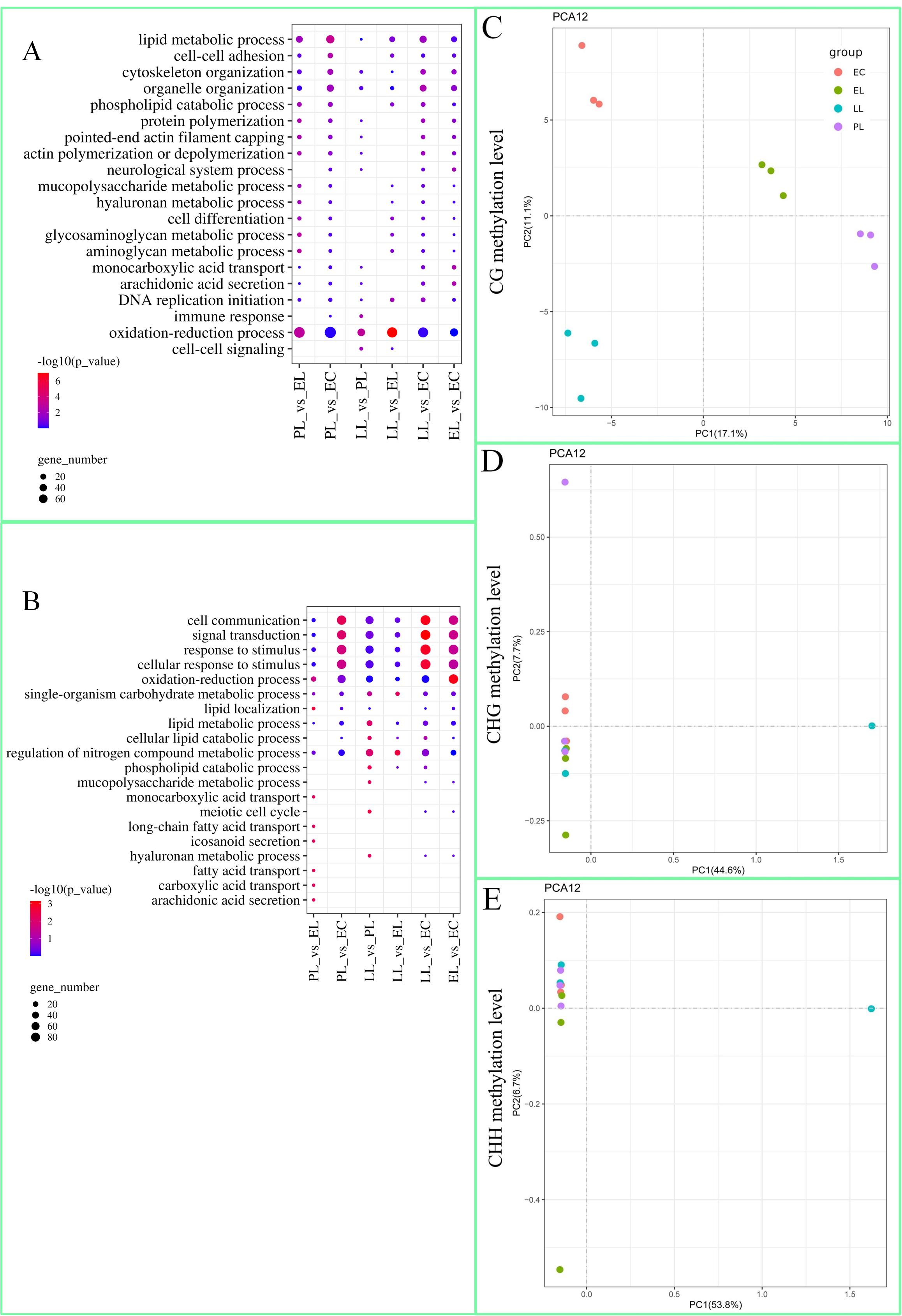

Supplement: Supplementary file 1 [file cimb-47-00331-s001.zip › Supplementary Figure S2.jpg]
